# Construction of anthropomorphic phantoms for use in dosimetry studies

**DOI:** 10.1120/jacmp.v10i3.2986

**Published:** 2009-08-06

**Authors:** James F. Winslow, Daniel E. Hyer, Ryan F. Fisher, Christopher J. Tien, David E. Hintenlang

**Affiliations:** ^1^ Department of Nuclear and Radiological Engineering University of Florida Gainesville Florida USA 32611‐8300

**Keywords:** anthropomorphic phantom, tissue‐equivalent material, organ dose, dosimetry

## Abstract

This paper reports on the methodology and materials used to construct anthropomorphic phantoms for use in dosimetry studies, improving on methods and materials previously described by Jones et al. [Med Phys. 2006;33(9):3274–82]. To date, the methodology described has been successfully used to create a series of three different adult phantoms at the University of Florida (UF). All phantoms were constructed in 5 mm transverse slices using materials designed to mimic human tissue at diagnostic photon energies: soft tissue‐equivalent substitute (STES), lung tissue‐equivalent substitute (LTES), and bone tissue‐equivalent substitute (BTES). While the formulation for BTES remains unchanged from the previous epoxy resin compound developed by Jones et al. [Med Phys. 2003;30(8):2072—81], both the STES and LTES were redesigned utilizing a urethane‐based compound which forms a pliable tissue‐equivalent material. These urethane‐based materials were chosen in part for improved phantom durability and easier accommodation of real‐time dosimeters. The production process has also been streamlined with the use of an automated machining system to create molds for the phantom slices from bitmap images based on the original segmented computed tomography (CT) datasets. Information regarding the new tissue‐equivalent materials, as well as images of the construction process and completed phantom, are included.

PACS number: 87.53.Bn

## I. INTRODUCTION

Anthropomorphic phantoms constructed from tissue‐equivalent materials have historically been used to provide a physical representation of the body's anatomy and attenuation characteristics for radiation dosimetry studies. Of particular interest for this publication is the use of anthropomorphic phantoms for measuring dose in diagnostic imaging procedures, where such measurements have been used by several authors to calculate average organ doses as well as effective dose in computed tomography (CT), cone‐beam CT, and pediatric radiology.^(^ ^1^ ^–^ ^3^ ^)^ Quantifying organ doses in physical phantoms offers a distinct advantage over computational methods because knowledge of the exact photon energy spectrum or irradiation geometry is not required. This is especially useful considering the increasing use of proprietary scanning techniques that are difficult to model, such as automatic tube current modulation in CT and automatic exposure control (AEC) in fluoroscopy. The majority of organ dose studies in diagnostic imaging utilize commercially available anthropomorphic phantoms such as RANDO (The Phantom Laboratory, Salem, NY) or ATOM phantoms (Computerized Imaging Reference Systems, Inc, Norfolk, VA). In order to provide a representation of the human anatomy, these commercially available phantoms typically use three tissue equivalent materials imitating bone, lung, and soft tissue. To allow access to organ locations for the placement of dosimeters, the RANDO and ATOM phantoms are assembled in axial slices 2.5 cm thick. Unfortunately, the widespread clinical use of these phantoms has been limited by their prohibitive costs.

The University of Florida (UF) has recently developed a series of low‐cost tissue‐equivalent materials that are easily prepared in the laboratory, and incorporated them in several sophisticated anthropomorphic phantoms. To date, this process has been used to create a series of three adult phantoms. Expanding upon methods originally published by White et al.,^(^ ^4^ ^,^ ^5^ ^)^ and later improved upon by Jones et al.,^(6)^ three tissue‐equivalent materials were developed for use in phantom construction: soft tissue‐equivalent substitute (STES), lung tissue‐equivalent substitute (LTES), and bone tissue‐equivalent substitute (BTES). BTES is based on an epoxy resin that forms a hard thermoset polymer, as previously described by Jones et al. STES and LTES are based on a new urethane mixture that forms a pliable compound. This material was chosen, in part, for ease in phantom construction, improved phantom durability, and easier accommodation of real‐time dosimeters.

The advantages of the UF phantoms compared to commercially available phantoms are that they utilize a 5 mm slice thickness, allowing greater options for dosimeter placement when performing internal dose measurements, and the anatomy is precisely known with respect to the CT data set used to construct the phantom. In addition, each physical phantom has a corresponding segmented computational phantom that was created from the same original CT data set, such as those developed by Lee et al.^(7)^ This allows the physical phantom to serve as a direct comparison to the computational phantom for the experimental validation of Monte Carlo codes. In turn, the computational phantom can be used to determine point‐to‐organ dose scaling factors, allowing the calculation of average organ doses from simple point organ dose measurements made in the physical phantom.^(8)^

The full‐body data set includes over three hundred axial slices; however, the lack of radiosensitive organs in the legs justified their exclusion from fabrication. As such, each phantom includes approximately two hundred axial slices, ranging from the crown of the head to mid‐thigh. All internal organs in the phantoms are modeled as soft tissue and, therefore, dosimeter placement for organ dose measurements is based solely on position of the segmented organs in the original data set. To aid in dosimeter placement, organ locations have been transferred onto each slice from full‐scale printouts of the original segmented data set.

## II. MATERIALS AND METHODS

### A. Materials

The tissue‐equivalent substitutes used for this undertaking were developed with two goals in mind: 1) similar physical properties to human tissue, such as density and attenuation coefficients, and 2) ease of integration into the phantom manufacturing process. To meet these goals, new urethane‐based STES and LTES were developed.

The developed tissue‐equivalent materials were evaluated by measuring the material density and attenuation properties. The attenuation coefficient of the STES was evaluated by measuring the attenuation from multiple thicknesses of material using a narrow beam geometry generated by clinical radiographic unit. Additionally the Hounsfield Unit (HU) values were measured in the completed phantom using a Siemens Somatom Sensation 16‐slice CT scanner operated at a tube voltage of 120 kVp and employing an mA modulated exposure control. The average HU was determined from the selected regions of interest (ROI) using areas of approximately 10 cm^2^.

Density measurements of each sample were then taken utilizing Archimedes's principle. A cured sample of each material was weighed on a scale with 0.001 gram precision to find the dry mass, $m_{\text{dry}}$, of each sample. The samples were then weighed submerged in a beaker of de‐ionized water to find the wet mass, $m_{\text{wet}}$, of each sample. Using both these measurements, as well as the known density of the de‐ionized water, $\rho_{H_{2}O}$, the density of each sample was calculated using Eq. (1):

(1) $$\rho_{sample}=\frac{m_{dry}}{\left[\frac{m_{dry}-m_{wet}}{\rho_{H_{2}O}}\right]}$$

#### A. 1. Soft tissue‐equivalent substitute (STES)

A new urethane‐based STES was designed to match the X‐ray attenuation and density of human soft tissue within the diagnostic energy range (80–120 kVp). Specifically, the STES was designed to have a density similar to that of human soft tissue $\left(1.04\;g/{\text{cm}}^{3}\right)$ and to achieve a target X‐ray attenuation coefficient based on the ICRU‐44 reference soft tissue composition.^(^ ^9^ ^,^ ^10^ ^)^ The commercially available, two‐part urethane rubber compound “PMC 121/30 Dry”, (Smooth‐On, Easton, PA), was combined with 2.8% by weight of powdered ${\text{CaCO}}_{3}$ (Fisher Scientific, Hanover Park, IL) to achieve these design goals. The calcium carbonate was added to the two parts of urethane and mixed with an electric mixer, with care being taken to ensure a homogeneous mixture with no undissolved ${\text{CaCO}}_{3}$. The durable, readily available urethane‐based compound was found to be easy to work with and did not suffer from phase separation problems frequently encountered with epoxy resin based STES. An additional benefit of the urethane‐based STES is its filexibility, which allows easy removal from molds after curing.

Adipose tissue was not specifically modeled in the construction of the anthropomorphic phantom. The distribution of subcutaneous as well as intra‐abdominal adipose tissue was initially determined to be too complicated to directly model with a specific tissue‐equivalent material. Thus, the STES was developed to be a homogeneous soft tissue analog that comprises skeletal muscle as well as organs, connective tissue, and adipose tissue.

#### A. 2. Lung tissue‐equivalent substitute (LTES)

Anew LTES was designed by combining uncured urethane‐based STES, prepared as described above, along with poly‐fil polystyrene micro beads (Fairfield Processing, Danbury, CT) in a 10:1 ratio by weight. This LTES is very uniform and permits the fabrication of a range of tissue densities spanning various levels of inspiration. Since it does not rely on a tissue surfactant and foaming agent, the LTES is more uniform and reproducible than the method proposed by White et al.^(11)^ While the density of lung tissue can vary widely depending on the level of inspiration, patients undergoing diagnostic procedures are typically asked to hold their breath during the exposure. Therefore, a value of $0.33\;g/{\text{cm}}^{3}$ was chosen for the LTES, representing the density of a fully inspired lung.^(10)^

#### A. 3. Bone tissue‐equivalent substitute (BTES)

The BTES used was the epoxy resin based material previously developed by Jones et al.^(6)^ By mass, the mixture of the BTES is as follows: 36.4% Araldite GY6010 and 14.6% Jeffamine T‐403 (Huntsman Corp., Woodlands, TX), as well as 25.5% Silicon dioxide and 23.5% Calcium carbonate (Fisher Scientific, Hanover Park, IL). It was designed to represent a homogenous mixture of cortical and trabecular spongiosa (bone trabeculae and bone marrow). The BTES composition was adjusted to match the mass density, mass attenuation coefficients (μ/ρ), and mass energy absorption coefficients $\left(\mu_{en}/\rho \right)$ for those defined by the Oak Ridge National Laboratory (ORNL) stylized model series^(12)^ within the diagnostic photon energy range. The effective atomic number for the BTES (8.80) is very similar to that of the ORNL reference tissue (8.59), and it was shown that values of μ/ρ and $\mu_{en}/\rho$ for BTES had a maximum deviation from ORNL reference values of only a few percent.^(6)^

### B. Phantom construction methodology

Initially, the methodology described by Jones et al.^(13)^ in the construction of a newborn phantom was to be used in the construction of the adult phantom series. This method involved several steps: preparing epoxy based soft tissue material in a vacuum chamber to eliminate air bubbles, pouring the material into a square mold, milling out the outer slice contour as well as appropriate voids for bone and lung tissue‐equivalent material, and, finally, filling these voids with bone or lung tissue‐equivalent material, as required. However, the far greater number and size of slices needed to construct an adult phantom, as compared to a newborn phantom, required many changes in the original construction methodology. The construction of the first adult phantom began with a segmented CT data set and an automated machining system and software (VisionPro Version 7, Vision Engraving and Routing Systems, Phoenix AZ), which was intended to speed up the phantom construction process. Once the phantom construction was initiated, problems were identified and overcome as they arose. The final means of production are detailed below.

#### B. 1. Using segmented tomographic images with the engraving system

As previously mentioned, three different phantoms have been constructed to date. The first phantom was based on a 35‐year‐old Korean adult male, 172 cm in height and 68 kg in total body weight.^(7)^ The exam was performed in conjunction with a cancer screening protocol using a Siemens Somatom Emotion Duo PET/CT system with a slice resolution of 1 mm. The next two phantoms constructed were based on hybrid computational phantoms of a 50th percentile adult male and female developed at the University of Florida. These phantoms originated from tomographic data, but were subsequently modified to match anthropometric dimensions and organ masses as defined by the International Commission on Radiological Protection (ICRP) publication 89^(14)^ reference data for a 50th percentile human in a process similar to that described by Lee et al.^(^ ^15^ ^,^ ^16^ ^)^ The original tomographic data for each hybrid phantom came from a 36‐year‐old Korean adult male (176 cm height, 73 kg weight) and 25‐year‐old adult female (163 cm height, 60 kg weight). The adult male exam was performed as part of a cancer screening protocol using a Siemens Somatom Emotion Duo PET/CT system with a slice resolution of 3 mm. The adult female was performed with a 4.5 mm slice resolution. All scans were performed at full inspiration with an in‐plane matrix size of 512×512pixels. Organ segmentation was performed manually under supervision of a radiologist. While approximately 100 different tissues were segmented in the computational data set, only the organs needed for the calculation of effective dose, as outlined in ICRP 103,^(17)^ were transferred to the physical phantoms.

The first step in constructing the phantom was to convert the segmented data set into a form that could ultimately be read with the automated machining software. Using ImageJ software (Version 1.34s, National Institute of Health, Bethesda, MD), each segmented image was converted into a bitmap representing only soft tissue and other tissues (bone, lung, air). This was accomplished by segmenting bone, lung, and air to a single pixel value representing “voids,” while all remaining soft tissues were shaded with another single value representing soft tissue. Registration marks for assisting in phantom assembly and alignment were also added to each bitmap image and the finished bitmaps were then imported into the VisionPro software. Each bitmap was adjusted to conform to the 256 value color range in the VisionPro software and vectorized in order to smooth the pixilated edges of the digital images. A speckle filter was used to eliminate tissue islands less than four pixels in area. Once these steps were complete, engraving paths for all areas represented by the soft tissue pixel value were then created for each slice. Realizing that smaller diameter “end mill” bits allow finer details to be cut, a 1/8‐inch diameter bit was selected for body engraving paths while a 1/16‐inch diameter bit was chosen for engraving paths in more detailed regions of the head.

**Figure 1:**
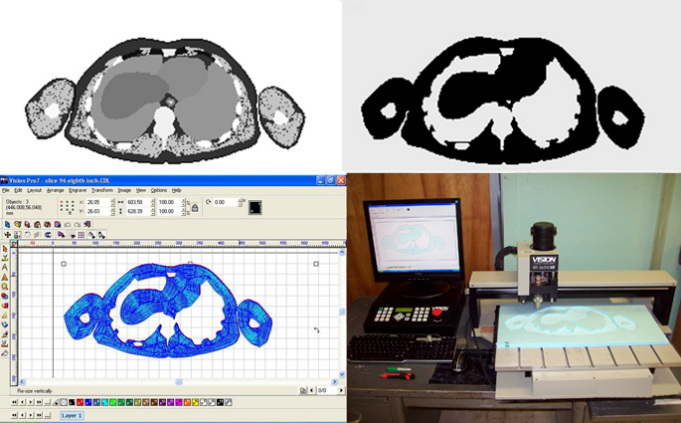
The steps in the phantom construction process: (top left) segmented CT image; (top right) soft tissue bitmap; (bottom left) VisionPro engraving path; (bottom right) engraving system milling a soft tissue mold.

The engraving paths were used to mill soft tissue molds in a high‐density foam, which could then be filled with the soft tissue substitute. Foam blanks were fastened to the engraving table and single‐pass engraving paths were set with depths resulting in 5 mm thick soft tissue slices. To create clean edges in each foam mold, a perimeter engraving path was first performed at a slow feed (0.6” per second), outlining the entire perimeter of the area to be cut. This was followed by a much faster rate fill engraving path (3” per second), which removed all foam material within the perimeter engraving path. Molds for each slice could be created in approximately ten minutes. The process of manufacturing a soft tissue mold is shown in Fig. 1.

After engraving was completed, the molds were checked to ensure that all areas to be filled with STES were connected, to aid in future placement. In cases where an area to be filled with STES was surrounded by bone or lung, small grooves were cut in the mold with a razor blade in order to connect the soft tissue island to the main body of the slice. This is similar to a stencil where the center of the letter “O” must be joined with thin connectors to ensure proper orientation. Finally, the job time for each slice was recorded. The job time and feed rate were used to determine the approximate volume/weight of soft tissue‐equivalent material needed for each slice.

#### B. 2. Fabrication of soft tissue

Depending on how many soft tissue molds were being filled at a time, an appropriate amount of the urethane‐based STES was mixed and immediately poured into the soft tissue molds. This was done fairly rapidly (less than 30 minutes), as the STES began setting immediately. The filled molds were covered with waxed paper and any trapped air pockets were relieved by slicing the waxed paper with a razor blade. The molds were then covered with smooth, weighted boards in order to force excess STES out of the molds, which would allow the soft tissue slices to cure at the correct thickness (5 mm). After roughly three hours, the weight and waxed paper were removed from the partially cured soft tissue slices. It is important to remove the waxed paper prior to the STES fully curing in order to facilitate removal. After 24 hours, the soft tissue slice could be removed from the mold and any excess STES around the edges was trimmed with a razor blade.

#### B. 3. Fabrication of lung inserts

For images that included lung tissue, separate molds were created in a similar fashion to the soft tissue molds described above, in order to produce lung inserts for the phantom. Unlike the STES, the LTES is not fluid and must be spread into the lung molds. As with STES introduction, waxed paper along with smooth, weighted boards were used to ensure that the LTES inserts were uniform in thickness. The LTES is not as strong as the STES and did not remove as easily from the foam molds, requiring that the molds be cut away from the newly formed lung slices. These slices, which were an exact fit to the corresponding voids in the soft tissue slice, were then fixed to the soft tissue slice with the introduction of the BTES into rib locations.

#### B. 4. Fabrication of bone

**Figure 2:**
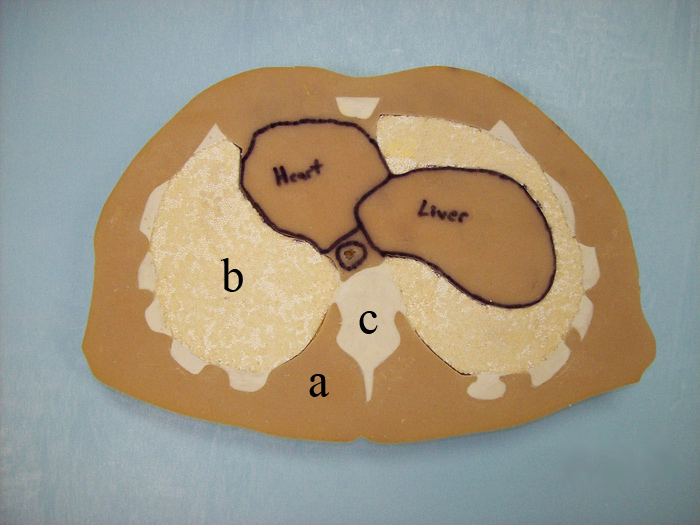
A fully formed phantom slice including: (a) STES; (b) LTES; and (c) BTES.

The method of placing bone into the soft tissue slices was analogous to that of Jones et al.^(13)^ First, the bottom of each soft tissue slice was sealed using contact paper to prevent any uncured BTES from running under the slice. Any soft tissue island connectors were then removed using a razor blade. An appropriate amount of BTES was mixed to fill the voids in the soft tissue slices that were left for bone tissue. A heat gun was used to warm the BTES material in order to reduce its viscosity and make it easier to mix and pour. The BTES material was then placed in a pastry bag including a pastry tip (#12) and forced into the appropriate voids in the soft tissue slices, taking care to avoid creating any air pockets during the pouring. Air pockets that were trapped during bone insertion would typically rise to the surface, where they could be pierced and eliminated. Bone locations were slightly overfilled because it was found to be easier to remove excess bone than to add additional bone after curing. The segmented data set was referenced to avoid accidentally filling any voids intended to contain air. The BTES was allowed to cure for 48 hours. Finally, the contact paper masks were removed and the bone locations within each phantom slice were sanded flush with the soft tissue using a belt sander with an 80 grit belt. Figure 2 shows a completed slice which includes the STES, LTES, and BTES materials integrated into a transverse slice of the phantom.

#### B. 5. Phantom assembly

Once all the phantom slices were completed, the organs and locations of dosimetric importance were selected. Full‐scale printouts of the segmented images containing these measurement locations were used to trace and label the organs of interest onto the physical phantom slice using a permanent marker. Additionally, phantom slices containing these locations were left unattached to a bordering slice in order to allow access for dosimeter insertion. All other slices were bonded to adjacent slices using commercially available wood glue. The glue was placed uniformly over all areas of a slice surface with the exception of air spaces and LTES. Wood glue has been found to behave radiologically similar to soft tissue at diagnostic energies.^(13)^ Bonding slices of the phantom into sections permits easy disassembly/reassembly of selected portions of the completed phantom. During assembly, slices were aligned using registration marks and then glued together sequentially. After assembly was completed, excess wood glue was removed using wire cutters and registration marks were removed with a razor blade.

## III. RESULTS

### A. Materials

#### A. 1. Soft tissue‐equivalent material

The STES was empirically evaluated using an X‐ray source (3.9 mm Al HVL at 80 kVp) to have an HVL of 25 mm at 80 kVp, and 29 mm at 120 kVp. The measured density was $1.04\;g/{\text{cm}}^{3}$. The average HU for the STES material was found to be 9.8, at the lower end of the widely accepted range for human muscle (10–40 HU). However, the measured value is considered acceptable because STES represents a homogenized mixture of both muscle and adipose tissue, with the latter having a HU range of −50 to −100.

#### A. 2. Lung tissue‐equivalent material

The density of the LTES was measured to be $0.33\;g/{\text{cm}}^{3}$, agreeing with the targeted lung density for full inspiration. The average HU for the LTES material is −678.4, consistent with widely accepted HU values for lung, which range from −500 to −1000.

#### A. 3. Bone tissue‐equivalent material

The BTES has been previously characterized^(6)^ and empirically evaluated to have an HVL of 9.8 mm at 80 kVp, and 13.3 mm at 120 kVp. The BTES material had an average HU of 622. This result is consistent with widely accepted HU values of bone, which range from 400 to 1000.

### B. Completed Phantom

**Figure 3:**
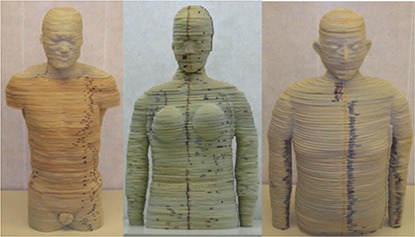
(a) Phantom based on a segmented CT data set of an adult male; (b) phantom based on a computational adult female hybrid data set; (c) phantom based on a computational adult male hybrid data set.

To date, three adult phantoms have been created using the methods and materials described in the previous section. As previously mentioned, the first phantom created, shown in Fig. 3(a), was an adult male based on a segmented tomographic data set. The color differences observed between phantom regions occurs as a result of extended exposure of one of the pre‐mixed urethane mixture components to humidity; however, testing showed no radiological difference. This color variation is more apparent in the first phantom since it was constructed over a longer period of time. While the arms are not shown in the figure, they can easily be attached when the phantom is used for dosimetry measurements. Also, as previously mentioned, the next two phantoms created were based on computational adult hybrid phantoms developed at UF representing the 50th percentile adult male and female, as shown in Fig. 3(b) and 3(c), respectively. Although not pictured, both hybrid phantoms also include a pelvis section that extends to mid‐thigh. Surface markings seen on all phantoms (black markings) refer to slice number and were used during the assembly process to keep slices in order.

**Figure 4:**
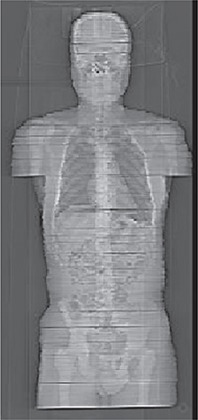
A CT topogram of a tomographic physical phantom.

Figure 4 shows a CT topogram of the adult tomographic phantom of Fig. 3(a). A Vac Fix reusable patient positioning system for radiation therapy (S&S Par Scientific, Houston, TX) was used to hold the phantom and keep the slices together during imaging. The horizontal dark lines located within the phantom present in Fig. 4 are slight gaps resulting from the vacuum bag's inability to perfectly hold all sections of a supine phantom in place. However, dosimetric measurements for CT have shown little difference when these gaps are present. The weight of the completed phantom as shown in Fig. 4 is 54 kg.

## IV. DISCUSSION

The urethane‐based STES has numerous advantages over the epoxy resin based soft tissue substitute originally proposed by Jones et al.^(6)^ First, it is much less viscous than the epoxy resin soft tissue substitute, making it easier to pour into the foam molds. Once cured, it is easily removed from the foam molds; this is not the case with the epoxy resin materials. Additionally, it requires fewer modifying constituents than epoxy resin based tissue‐equivalents, and therefore better retains homogeneity. The urethane‐based STES remains pliable and strong when cured, while the epoxy resin soft tissue substitute is brittle when cured and can break under stress or when dropped. Because of these properties, the urethane‐based material is more durable and unlikely to be damaged with use. Finally, STES better accommodates the insertion of real‐time dosimeters, only requiring a thin slit to be cut into the material to allow passage of electrical or optical cords that connect the active regions of the detector to a read‐out device. This avoids any potential concerns about radiation streaming along machined dosimeter channels.^(13)^

Creating molds resulting in uniform 5 mm thick phantom slices proved more challenging than expected. Small variations in individual slice thickness can accumulate to create large discrepancies when hundreds of slices are combined. Early on, molds would occasionally display a variation in cutting depth throughout the slice. The engraving system hardware and software was initially suspected and investigated. However, it was found that this variation in cutting depth was the result of the foam template bowing upwards and losing adhesion to the engraving table during the milling process. Similarly problematic, engraving path depths were also initially set to the desired 5 mm, which was expected to result in a 5 mm thick soft tissue slice. However, the excess freshly poured STES could not be pressed infinitely thin, and so an additional thickness of 0.5–1 mm would often result. Thicknesses of this magnitude, reflexively considered minor, are in fact considerable with respect to 5 mm thick slices, resulting in slices that were 10%–20% too thick. This problem was corrected by adjusting the indicated engraving depth to 4 mm and using a consistent procedure to define the cutting surface to the engraving system.

## V. CONCLUSIONS

A unique methodology has been developed to construct anthropomorphic phantoms for use in dosimetry studies. While the value of this methodology has already been proven with the construction of three adult phantoms, it should be noted that the same methodology could be applied to the construction of phantoms of all sizes and ages. In particular, our group plans to develop a family of phantoms that accurately represent patients of differing heights and weights. Future works also include the investigation of an adipose tissue‐equivalent substitute which could be added to the existing phantoms – or included as an additional step in the construction of a new phantom – to represent subcutaneous fat, in order to accurately model more obese patients.

While anthropomorphic phantoms have many potential applications, this particular phantom series was created to quantify organ doses from diagnostic procedures. It is anticipated that other institutions could create their own phantoms for regular clinical use by following the methodology and using the described tissue equivalent materials for a total material cost of less than $3,500.

## ACKNOWLEDGEMENTS

This work was supported by the U.S. Department of Energy under project award number DE‐GF07–05ID14700, and the Center for Disease Control through TKC Integration Services TO81.
